# Design of Multifunctional Mesosphere-Ionosphere Sounding System and Preliminary Results

**DOI:** 10.3390/s20092664

**Published:** 2020-05-07

**Authors:** Tongxin Liu, Guobin Yang, Zhengyu Zhao, Yi Liu, Chen Zhou, Chunhua Jiang, Binbin Ni, Yaogai Hu, Peng Zhu

**Affiliations:** 1 School of Electronic Information, Wuhan University, Wuhan 430072, China; tongxin_liu@whu.edu.cn (T.L.); zhaozy@whu.edu.cn (Z.Z.); liuyiwhuhan@whu.edu.cn (Y.L.); chenzhou@whu.edu.cn (C.Z.); chuajiang@whu.edu.cn (C.J.); bbni@whu.edu.cn (B.N.); yaogaihu@whu.edu.cn (Y.H.); 2 Institute of Space Science and Applied Technology, Harbin Institute of Technology, Shenzhen 518000, China; 3 School of Information Science and Technology, Hunan Institute of Science and Technology, Yueyang 414006, China; zhupeng@whu.edu.cn

**Keywords:** mesospheric sounding, ionospheric sounding, pseudo-random phase-code, miniaturized antennas

## Abstract

This paper describes a novel sounding system for which the functions of the medium frequency (MF) radar and the ionosonde are integrated on the same hardware platform and antenna structure, namely the middle atmosphere-ionosphere (MAI) system. Unlike the common MF radar, MAI system adopts the pseudo-random (PRN) phase-coded modulation technology, which breaks the limitation of the traditional monopulse mode. Through the pulse compression, only a small peak power is needed to achieve the signal-to-noise ratio (SNR) requirement. The excellent anti-jamming performance is also very suitable for the ionospheric sounding. One transmitting and six receiving modes are adopted for the MF sounding. While neglecting the structure of the T/R switches, the coupling interference between the transmitter and the receiver may also be avoided. Moreover, by employing a miniaturized antenna array composed of progressive-wave antennas for the MF receiving and ionospheric sounding, the MAI system takes account of the requirements of the inversion algorithms of MF radar and the large bandwidth need for the ionospheric sounding concurrently. Such an antenna structure can also greatly simplify the system structure and minimize the difficulty of deployment. The experiments verified the availability of the system scheme and its engineering application significance. Through further analysis of the sounding data, the wind field of the mesosphere, the electron density of D layer and electron density profile from layers E to F were obtained at the identical location. The capability of MAI system can play an important role in studying the interaction and coupling mechanism between the mesosphere and ionosphere.

## 1. Introduction

The mesosphere and ionosphere are both important transitional regions of the Earth’s atmosphere. Among them, the mesosphere is defined by temperature gradients, located at 50–85 km, while the ionosphere is defined by the degree of atmospheric ionization, embedded in the middle of the thermosphere, generally referring to the partial ionization area above 60 km. Although they are defined in different ways, they are overlapped and coupled in height. As soon as the mesosphere is illuminated and ionized, it is also denoted as lower ionosphere. However, for the purpose of distinction, it is still referred to as “mesosphere” in subsequent statements of wind filed calculation in this article. Thus, it is very meaningful to promote the comprehensive, in-depth study of the structure and coupling mechanism between them by conducting these overlapped regions sounding compatibly [1]. However, current experiments are usually carried out separately with the equipment of different types.

For the sounding of the mesosphere, space-based sounding depended on the satellites, land-based ones like mesosphere-stratosphere-troposphere (MST) radar and medium frequency (MF) radar are the most commonly used devices. Among them, MF radar is the most widely used one, which can detect the atmospheric wind field and electronic density in the range of 60−100 km of the adjacent space, especially the mesosphere. The research on MF radar can be traced back to the work of Gardner and Pawsey [2]. In 1950s, the differential absorption experimental (DAE) algorithm was employed by them on MF radars to calculate the electron density of lower ionosphere [3]. Based on the spaced antenna (SA) technology and the full correlation analysis (FCA) algorithms proposed by Mitra and Briggs [4,5], MF radars were also beginning to be used for the estimation of the regional wind field of the mesosphere [6,7,8,9,10,11]. Compared to the space-based methods and MST radars, the structure of MF radar is relatively simple and easier to implement with comparatively less cost. Up to now, numerous MF radars have been built and put into routine operation around the world. The performance is also improving with the progress of the hardware and software technology. In 1975, Manson and Meek leaded to develop the advanced MF radar receivers and data processing methods for atmospheric dynamics studies in the mesosphere and lower thermosphere (MLT) region [12,13]. In 1994, the Yamagawa Radar was built in Japan and, based on the sounding data, Igarashi and Murayama et al. reported their coordinated observations of the dynamics and coupling processes of the mesosphere and lower thermosphere winds at the middle-high latitude in 1995 [14]. In 1995 the team led by Vincent and Reid built in Australia the world’s largest MF radar at Buckland Park, whose total transmitting power reaches 135 kW [15]. Saura radar, located in Northern Norway, is also very powerful and flexible. Relying on the large antenna array and larger transmitting power, Saura radar can be used for wind estimation, deriving electron denstity and the measurements of gravity wave momentum fluxes [16,17]. Although MF radar has many advantages, but at a lower operation frequency, the large mechanical dimensions of the commonly used standing-wave antennas also means a greater difficulty for construction. Moreover, due to the prevalent monopulse working mode, a quietly large transmission peak power is required, which makes the cost and power consumption huge. For instance, the length of the dipole antenna employed by Wuhan MF radar built in 2000 reach 75 m, with a peak power as high as 64 kW [18].

As a special type of radar, the development of ionospheric sounder has a long history and many mature devices have been developed [19]. Through continuous hardware improvements and the upgrades of the software, Dynasonde, developed by the National Oceanic and Atmospheric Administration (NOAA), and Digisonde, developed by University of Massachusetts Lowell, have become the currently most advanced ionosonde systems [20,21]. As early as the 1990s, the Ionospheric Laboratory of Wuhan University also began work on the development of the Wuhan Ionospheric Oblique Backscattering Sounding System (WIOBSS) to research the large region of the ionosphere in real-time [22]. By now, it has become a highly integrated, multi-functional platform with the abilities of the vertical, oblique, backscattering sounding and multi-station networking [23,24,25]. When some spaceborne methods are employed to probe the atmosphere, ionospheric observations can also be carried out using satellite networks. Masato Furuya detected midlatitude Sporadic-E (Es) relaying on the interferometric synthetic aperture radar (InSAR) in 2017, which clarified the spatial structure with unprecedented resolution [26]. Nina et al. also investigated the influence of the perturbed (by a solar X-ray flare) ionospheric D-region on the global navigation satellite systems (GNSS) and synthetic aperture radar (SAR) signals in 2019 [27]. Although the observation range is large in this way, the establishment of satellite networks is difficult. And mainly from the perspective of the total electron content (TEC), it is not easy to obtain the real-time ionospheric electron density profile of a specific location. Some other networks such as automatic packet reporting system (APRS) and automatic dependent surveillance broadcast (ADS-B) can also be used for ionospheric studies [28]. However, the same problems still remain. At present, the ionosondes are still difficult to replace for ionospheric sounding. Compared with MF radar, the ionosonde’s transmitting power and the size of the antennas are rather small. However, usually working in high frequency (HF) band, the vast majority of the existing ionosondes can only study the ionosphere above the E layer, which is usually higher than 100 km. This makes it applicable only to a relatively narrow mission scope, which may lead the observation and research to be unilateral.

In this paper, we propose a novel multi-functional system to satisfy the sounding of the mesosphere and ionosphere simultaneously, which is referred to as the middle atmosphere-ionosphere (MAI) system in this paper. The functions of MF radar and ionosonde are integrated on the same hardware platform and antenna array to achieve the complementarity with each other. Different from the traditional MF radars, the pseudo-random (PRN) phase-coded modulation technology is employed. Due to the extra gain generated by the pulse compression, the peak power required for transmission is greatly reduced. The excellent anti-jamming performance is also very suitable for ionospheric sounding. For the mesospheric sounding MAI system adopts a one-transmitting-and-six-receiving mode, which is unusual. While omitting the transceiver switches, the possible coupling interference may also be avoided. Moreover, innovatively, miniaturized two-wire progressive-wave antennas are employed on the MAI system to achieve the functions of MF echoes’ receiving and ionospheric sounding simultaneously [29]. The mesospheric parameter inversion algorithms and the wide band requirements for ionospheric sounding are taken into account concurrently. In addition to verify the feasibility of this antenna solution, it also greatly reduces the volume of the system and the difficulty of construction. Through the flexible switch between the working modes of the mesospheric and ionospheric sounding, the wind field of the mesosphere, the electron density distribution of the lower ionosphere, the ionogram and its inversion profile at the same location can be obtained. The acquisition of these parameters may have important value to the study of the interaction and coupling mechanism between the mesosphere and ionosphere.

## 2. System Description

**Figure 1:**
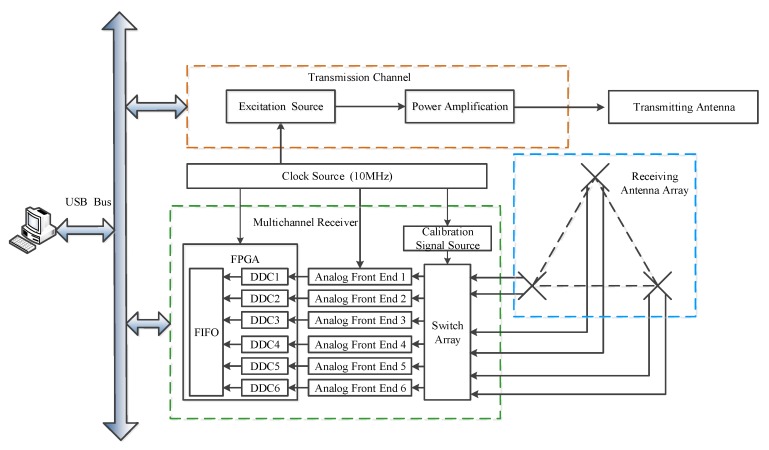
The overall structure of the MAI system, which is mainly divided into three parts: the transmitting channel, multi-channel receiver and receiving antenna array.

The overall structure of the MAI system is mainly composed of the transmission channel, the multichannel receiver and the antenna array. Among them, the transmission channel generates the sounding waveform. The sequence of the pseudo-random phase code is employed to modulate the transmitting waveform, which breaks through the monopulse working mode of the traditional MF radar. With the pulse compression of the complementary code and the gains of the coherent accumulation (pulse accumulation), the MAI system has strong anti-jamming and clutter suppression abilities, both of the mesospheric and ionospheric soundings. The multichannel receiver is responsible for analog processing and digital down-conversion (DDC) of the echo signals. In order to calibrate the amplitude and phase consistency between the receiving channels, an additional reference source is introduced into the device. The electrical length of the feeders connected to the receiver and antennas are strictly measured and adjusted to be equal. The method of these independent transceiver channels does not require high-performance transceiver switches, but also avoids the possible interference. A miniaturized designed antenna array is employed to meet the sounding and the analysis algorithms’ requirements, which has the advantages of small size and low cost. In order to adapt to the large bandwidth required for ionospheric sounding, progressive-wave antennas are used as the elements of the array. The system structure diagrams and the main technical indicators of MAI system are shown in Figure 1 and Table 1, respectively.

### 2.1. Transmission Channel 

**Figure 2:**
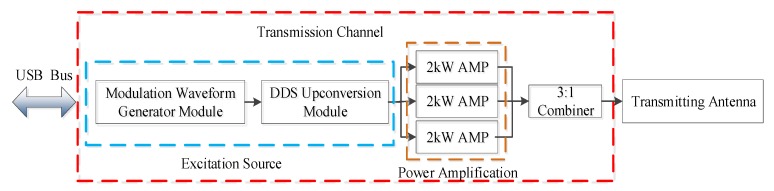
The transmission channel of MAI system which is mainly composed of the excitation source and power amplifier.

The transmission channel of MAI system is a single channel subsystem, as shown in Figure 2, which can be roughly divided into two parts: the excitation source and the power amplifier. Firstly, the baseband signal of pseudo-random coded modulation is generated by the waveform generation module according to the transmitting parameters. Then, after up-conversion by the direct digital synthesizer (DDS) device, the signals of about 1 mW are fed into three 2 kW broadband power amplifier (AMP) units. Finally, through a three-in-one combiner, the total power of 6 kW is fed into the transmitting antenna. 

**Figure 3:**
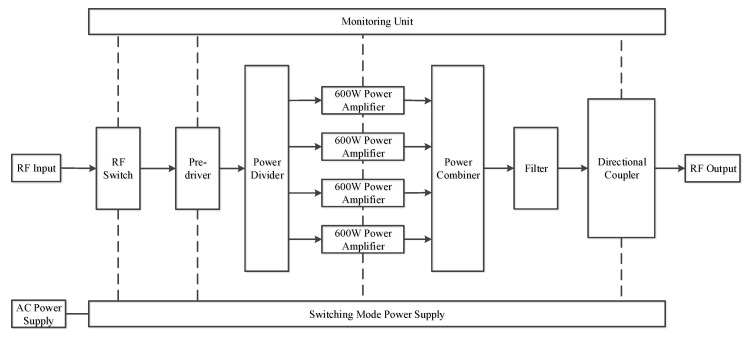
The block diagram of the power amplifier unit. 4 × 600 W linear amplifier can provide 2 kW transmission power.

The 2 kW broadband power amplifier unit adopts the all-solid-state structure, which is shown in Figure 3. It mainly consists of pre-driver, power divider, 4 × 600 W linear amplifier, and power combiner, directional coupler, monitoring unit and power supply apparatus. The monitoring unit monitors the output power and voltage standing wave ratio (VSWR) at all times. Once the singular changes occur, such as the over-voltage, over-current or the over-power events, the transmitter will be issued a turn-off command. When the fault is eliminated, the transmission channel resumes to work.

### 2.2. Sounding Waveform 

**Figure 4:**
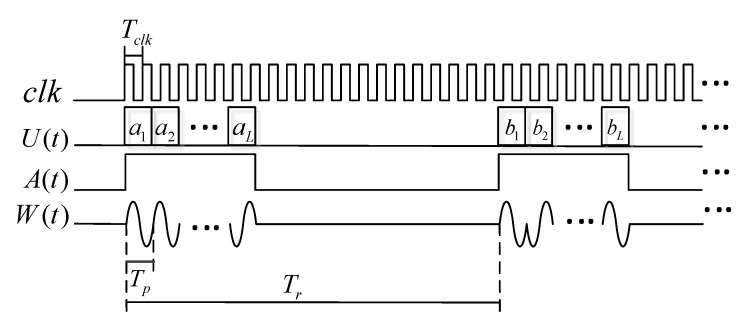
The specific waveform of the radar transmitting signal. Driven by the system clock clk, the transmitting waveform W(t) is generated according to the code sequence U(t) and transmitting pulse A(t). The positive and complement codes are transmitted alternately.

Breaking the limitation of the traditional monopulse mode, the interpulse coding waveform is applied to the MF sounding in MAI system. 16-bit bi-phase complementary sequences are selected as the pseudo-random modulation code for MAI system. The specific waveform of the transmitting signal is shown in Figure 4. 

In Figure 4, clk is the synchronized clock, with the period $T_{clk}$. U(t) represents the code sequence. The positive and the complement sequences are shown in Table 2. Further, 0 and 1, respectively, represent the phase 0 and π. The duration of each code bit is $T_{p}=12.8\;\mu s$. A(t) shows the time-sequence of the transmitting and the receiving. During the sounding period $T_{r}=320\times 12.8\;\mu s$, the sounding signal is transmitted firstly (when A(t) is in high level), while the remaining time is used to receive the echo signals (when A(t) is at low). W(t) is the actual sounding signal waveform.

**Figure 5:**
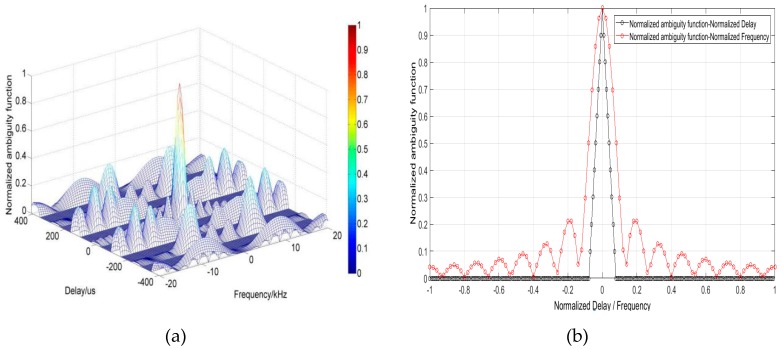
Normalized ambiguity function of complementary code: (**a**) Normalized ambiguity function; (**b**) Normalized ambiguity cut for Delay = 0 μs and Frequency = 0 kHz. It has a “pushpin type”ambiguity. And the section of 0 kHz has only one main lobe with no side lobe. Thus, it is very suitable for soft target sounding.

The concept of complement code is first proposed by Golay [30]. By using the feature of the zero autocorrelation sidelobe of a pair of complementary sequences, the complementary code can effectively eliminate the sidelobe interference in the sounding. Figure 5 is from the reference [25], showing the normalized ambiguity function graphs of the 16-bits sequences employed in MAI system. Figure 5a is the three-dimensional ambiguity function graph, Figure 5b shows the sections of zero Doppler and zero shift. Especially, the characteristics of its zero autocorrelation sidelobe intuitively. Obviously, as a kind of PRN sequence, the complementary code has a “pushpin type” ambiguity function, which means a high resolution in distance and speed and good sounding accuracy. It is very suitable for soft target sounding applications such as the mesospheric and ionospheric soundings investigated in this paper.

According to the waveform system, some specific radar parameters are determined. As the range resolution of bi-phase coded modulation waveform is determined by the duration of each code bit, this parameter of MAI system can be expressed as Equation (1) [31]:

(1) $$R_{\min}=\frac{cT_{p}}{2}=1.92\;\mathrm{km}$$

where *c* is the speed of light.

The maximum sounding range $R_{u}$ and the pulse repetition frequency (PRF) can be determined by Equations (2) and (3) [31]:

(2) $$R_{u}=\frac{cT_{r}}{2}=614.4\;\mathrm{km}$$

(3) $$PRF=\frac{1}{2T_{r}}=122\;\mathrm{Hz}$$

Due to the strong near-field coupling of the transmitting signal, there is a certain fade zone $R_{d}$. When L is defined as the bit length of the code sequences, $R_{d}$ can be determined by Equation (4) [31].

(4) $$R_{d}=L\cdot \frac{cT_{p}}{2}=30.72\;\mathrm{km}$$

By considering that the height range of the mesosphere or the lower ionosphere visualized by MAI system in the mesospheric sounding model is 70–100 km, and the concerned height range of the ionosphere is even higher, this interference of the fade zone will not cause any seriously adverse effects.

In addition, except for the Adelaide MF Rada and Saura radar with a large cross antenna array, whose average power are 240 W and 600 W [16,17,32], the average transmitting power of most MF radars is only about 100 W. However, in the case of MAI system, even though the peak transmitted power $P_{peak}$ is only 6 kW, the pulse coded waveform can generate a larger duty ratio ${}_{D}$, and the average power $P_{aver}$ can reach 300 W, as Equation (5) shows [31].

(5) $$D=\frac{LT_{p}}{T_{r}}=5\%,\;P_{aver}=P_{peak}D=300\;W$$

Also because of the monopulse mode and small duty ratio, for the traditional MF radars, despite the coherent accumulation, it is still quite difficult to obtain enough processing gains. Therefore, a large peak power is often required to ensure the signal-to-noise ratio (SNR) of the received echoes. Comparatively, complementary code can provide an additional correlation compression gain benefits from its good correlation characteristic. For ${}_{L}$-bit complementary code sequences $A=\left\{a_{n}\right\}$,$B=\left\{b_{n}\right\}$, the correlation function $R_{A}(\tau )$, $R_{B}(\tau )$ can be determined as Equation (6)

(6) $$\{\begin{array}{c} R_{A}(\tau )=\sum_{k=1}^{L}a_{k}a_{k+\tau },R_{B}(\tau )=\sum_{k=1}^{L}b_{k}b_{k+\tau }\\ R_{A}(\tau )+R_{B}(\tau )=\{\begin{array}{c} 2L,\tau =0\\ 0,else \end{array} \end{array}$$

Therefore, a pair of 16-bit sequences will bring compression gain of 12 dB. After a certain number of coherent accumulations, the processing gain would reach more than 20 dB, which makes it possible to obtain a higher SNR at a certain receiving threshold level for both the mesospheric and ionospheric sounding.

### 2.3. Multichannel Receiver

The receiving subsystem of MAI system is a 6-channel digital intermediate frequency (IF) receiver. After filtering, amplifying, mixing, and A/D sampling at the analog front-ends, the six digital IF signals are input into the DDC module in parallel. Through the digital IF processing for demodulation at the DDC module, the baseband signals are uploaded via USB bus for further analysis. The algorithms of the pulse compression and the physical parameter inversions of the baseband signals are realized by software on the host computer.

**Figure 6:**
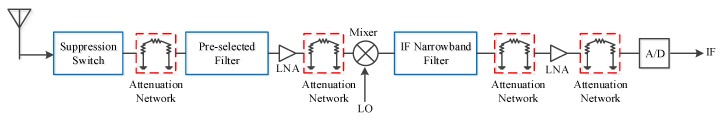
The circuit design of the analog channel. Between the levels, several “π” networks are employed to match the impedance and adjust the gain consistency of each channel.

The circuit design of each analog channel is shown in Figure 6. A suppression switch isolates the fade zone through timing control. And the multi-channel suppression switches constitute the switch array to suppress the near field coupling of the transmitting antenna during the transmission. Considering the need of MAI system to ensure the receiving performance of both MF and HF bands, the preferred filter of the first stage chooses a larger bandwidth, which is 0–30 MHz, covering the whole working frequency band. Two-stage amplification mode is employed in this system. The first low noise amplifier (LNA) is placed before the mixer, while the second stage is an IF amplifier. The total gain of the front-end can reach 53.1 dB. In order to effectively suppress the mirror frequency, the intermediate frequency is selected at a higher frequency band of 71.4 MHz.

As the range resolution of the MAI system is 1.92 km, corresponding to the duration of each code bit $T_{p}=12.8\;\mu s$, the IF bandwidth B of the radar is selected as Equation (7) [31]:

(7) $$B=1.5/T_{p}=117.1875\;\mathrm{kHz}\approx 120\;\mathrm{kHz}$$

To realize the impedance matching of two-port network between the different levels, “π” resistance networks with an attenuation value of 1dB are added. Therefore, when the filter insertion losses are included, the total noise figure (NF) of each analog front-end is about 10 dB. Then the sensitivity of the analog front end can be determined as Equation (8) [31]:

(8) S=−114 dBm+NF+10logB(MHz)≈−113 dBm

For the MAI system, the selected sampling digit of ADC is N=14 bits with a $2.5\;V_{pp}$ full voltage range. When the sampling rate is $f_{s}=20\;\mathrm{MHz}$, the maximum SNR of ADC can be calculated as Equation (9) [31]. Further, the dynamic range DR can be determined as Equation (10), which can also be considered as the dynamic range of the receiver [31].

(9) $$SNR(dB)=6.02N+1.76+10\log(f_{s}/2B)=105.2\;\mathrm{dB}$$

(10) $$DR=V_{\max}/V_{\min}=77.5\;\mathrm{dB}$$

where $V_{\max}$ and $V_{\min}$ represent the maximum sampled voltage and voltage resolution of ADC, respectively.

**Figure 7:**
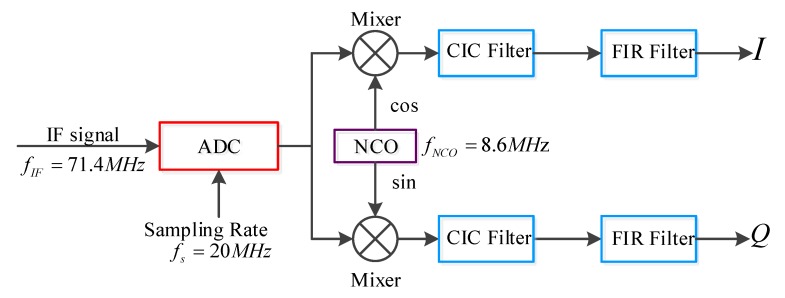
The structure of the DDC module. Digital mixing and filtering are implemented in FPGA, which generate the baseband signals and reduce the data rate.

In the DDC module, as shown in Figure 7, the 8.6 MHz orthogonal signals are generated by the numerically controlled oscillator (NCO) for digital mixing. Through the cascade integrator comb (CIC) and finite impulse response (FIR) filters, the echo signals are converted into the baseband I/Q data. The simple structure of CIC filter reduces the difficulty of design [33], and FIR filter compensates for the irregularity of CIC filter’s passband. Due to the 256 times downsampling during the process of the two-stage anti-aliasing filters, the output data rate of the digital signal of each channel is reduced from 40 Mbps to 156.25 kbps. The whole DDC module is implemented in a field-programmable gate array (FPGA) chip for easy modification and high-speed operation.

### 2.4. Miniaturized Antenna

The antenna of MAI system should be adapted to the mesospheric and ionospheric sounding mode simultaneously, which means that the antennas need to guarantee the performance at MF band while ensuring that the serious beam splitting and directivity change would not occur at HF band. Apparently, the large-sized standing-wave antenna array employed in conventional MF radar is not suitable for the MAI system. Therefore, two schemes are adopted in this paper for the functions of MF radar and the ionospheric sounding. Moreover, some simulations are also carried out based on computer simulation technology (CST).

**Figure 8:**
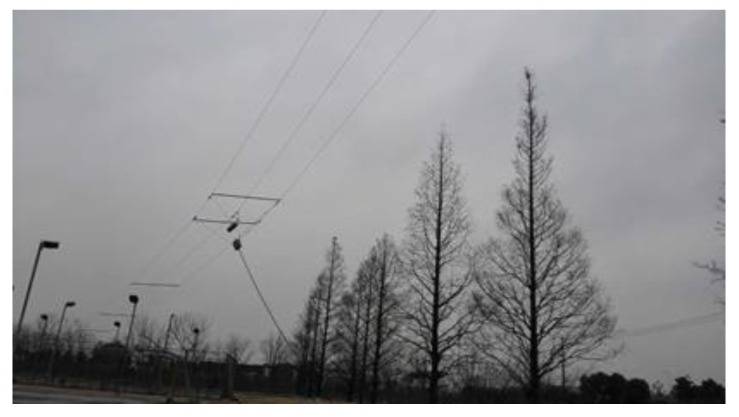
Erection image of 75 m three-wire transmitting antenna. It is a linear polarized dipole with the feed point in the center.

For the mesospheric sounding, MAI system adopts the mode of one transmitting and six receiving. A 75 m three-wire linear polarized dipole antenna is employed to match the MF band. The physical erection image is also can be seen as Figure 8. The frequency of 1.98 MHz is chosen as the working point for the mesospheric sounding. The simulation results of the variation of the transmitting characteristics with the erection height at 1.98 MHz are shown in Table 3. And the impedance is 50 Ω. When the erection height is within 6 m, the directivity gain increases with height slightly, but when it exceeds 4 m, the VSWR deteriorates rapidly. Obviously, the height of 3 m is the most suitable choice. It has the best VSWR to guarantee the radiation characteristic, and the lower erection height also reduces the construction difficulty. 

**Figure 9:**
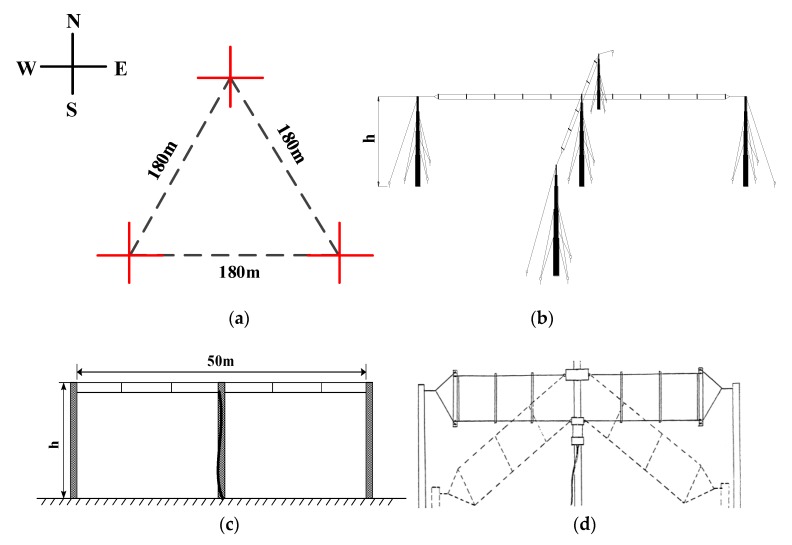
The antenna erection form: (**a**) The array structure, with a positive triangle structure; (**b**) The erection form of each vertex. The two antennas are erected in an orthogonal way; (**c**) The schematic erection form of a single receiving antenna with the feed point in the center. Due to the influence of gravity, the antenna cannot be straightened completely. The distance between the two poles is 50 m; (**d**) The structure image of the Barker & Williamson Broadband Folded Dipole Antennas. The dotted line indicates the erection scheme type of the inverted “V”, which is not adopted in this paper.

For the MF echoes’ receiving, MAI system employs two-wire dipole progressive-wave Barker & Williamson broadband folded dipole antennas [34], the length of which is 54.9 m, compared with Wuhan MF radar, it is reduced by 20 m. When MAI system works in SA mode at 1.98 MHz, an equilateral triangle array with a side length of 180 m is employed for receiving. At each vertex of the array, two antennas are crossed horizontally in an orthogonal polarization state. The specific receiving antenna erection shape is shown in Figure 9. The nearest one is 200 m away from the transmitting antenna. Figure 9a is the array structure and Figure 9b is the erection form of each vertex. In this way, although there is only one transmitting channel, the separate reception of the ordinary wave (O) and the extraordinary wave (X) can still be achieved. Figure 9c shows the schematic erection form of a single receiving antenna and Figure 9d shows the structure image. The erection height is indicated by h. Due to gravity, the antenna cannot be completely straightened, so the spacing between poles is only 50 m.

Further, Table 4 shows the variation of the receiving characteristics with the elevation height of the receiving antennas. In the range of 4–25 m, the directivity gain has little change. However, when the erection height is above 12 m, the beamwidth expands gradually. Considering the difficulty of the construction, the 8 m height for erection is a suitable choice. 

**Figure 10:**
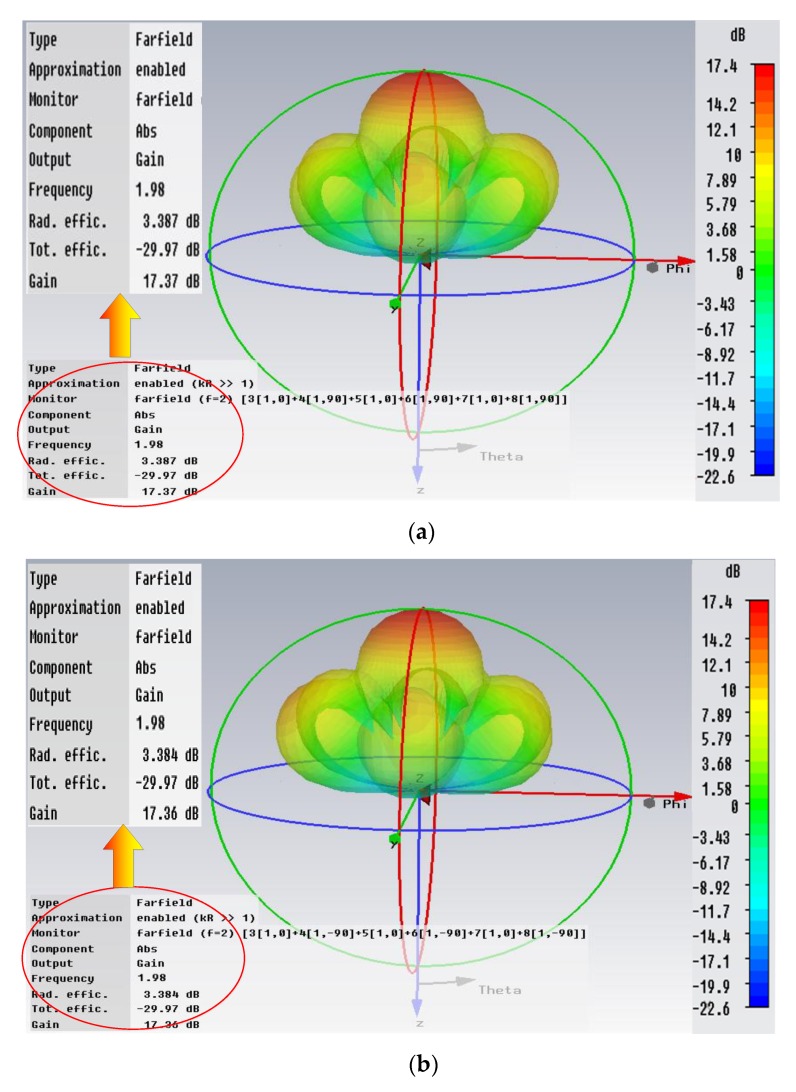
The pattern of the antenna array with the impedance of 50 Ω: (**a**) O-mode wave; (**b**) X-mode wave. No matter for O or X-mode wave, the antenna array has a high gain and vertical upward main lobe.

Thus, the whole array pattern simulation results based on CST can be obtained as Figure 10. Figure 10a,b shows the simulation results of O and X waves, respectively. The gain of the whole array is above 17 dB. The synthetic beam-pointing is vertical upward with a 27.4° beamwidth. The simulation results prove that the antenna array design scheme of this paper has a good receiving characteristic and can effectively meet the requirements of the mesospheric sounding with strong feasibility and operability. The whole array is characterized by high gain, narrow beamwidth, low cost and small structural size. Compared with the common MF radars, whose antenna towers are usually higher than 20 m, or use of the digital beam forming (DBF) antenna array with dozens of antennas, the MAI system employs only several 8 m towers and six small size progressive-wave receiving antennas. 

**Figure 11:**
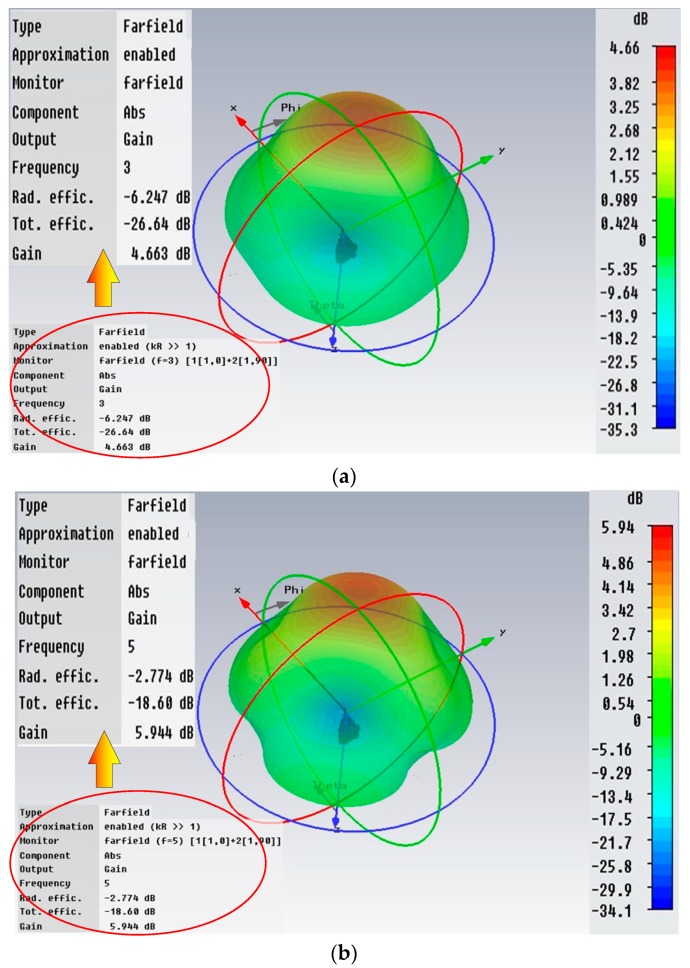

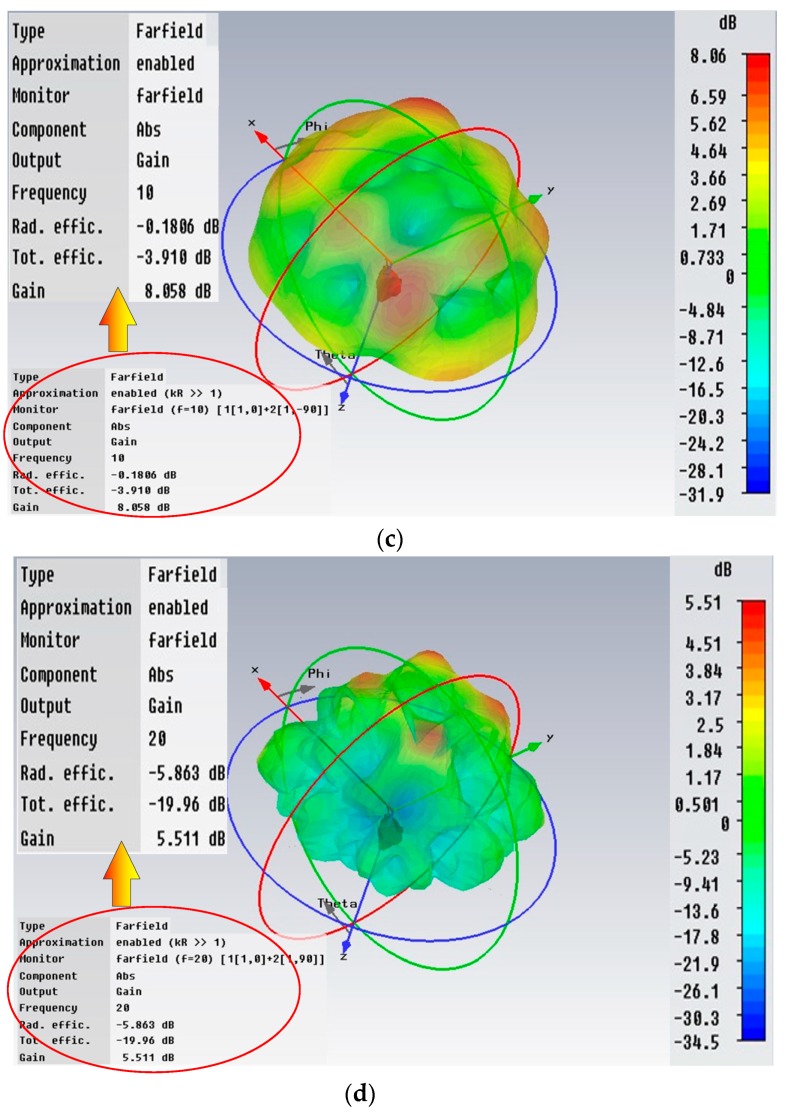
The pattern of the signal antenna in HF band with the impedance of 50 Ω: (**a**) 3 MHz; (**b**) 5 MHz; (**c**) 15 MHz; (**d**) 20 MHz. Although the main lobe splits as the frequency increases, it is not important for ionospheric sounding. The gain of full working band is more than 4 dB and the pointing direction is always upward.

When the MAI system works for ionospheric sounding, the mode of one transmitting and one receiving is usually adopted in order to reduce the amount of data and the processing time. For both of the transmitting and receiving, the 54.9 m progressive-wave antennas are employed to adapt to the wide frequency bandwidth. Switching from the mesospheric sounding mode to ionospheric mode only requires changing the antenna interface at the host device. The simulation results of the antenna’s performance in HF band is shown in Figure 11. Figure 11a–d represent the cases of 3, 5, 10, and 20 MHz. Though the main lobe splits at the higher frequency band, but for the ionospheric sounding which requires less stringent directivity, it can still meet the need. The gain of full working band is more than 4 dB and the pointing direction is always upward.

## 3. Prototype Device

**Figure 12:**
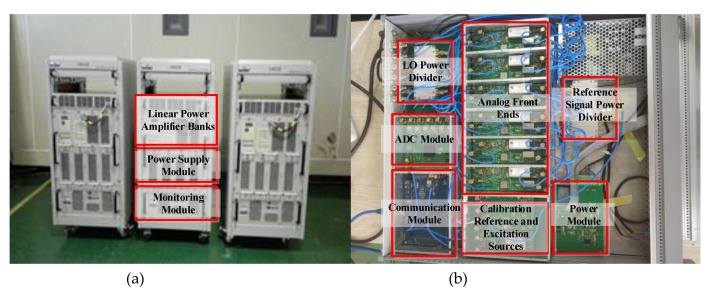
The finished product of the prototype: (**a**) 2 kW power amplifier units. Three units together provide 6 kW for transmitting. (**b**) The transmitting excitation source and multi-channel receiver. The aluminum alloy frames are shielded between the modules.

In order to verify the correctness of the scheme described in this paper and the value of engineering practice, we have designed and developed a prototype device. The finished product shown in Figure 12a shows three 2 kW power amplifier units. For every unit, the main components, i.e., the monitoring module, power supply module and linear power amplifier, are placed independently in the frame of three layers of the same movable cabinet, which is convenient for disassembly, reorganization and troubleshooting. In Figure 12b, the transmitting excitation source and multi-channel receiver are integrated into the same casing. Each module, especially the receiving channel, is shielded and isolated by an aluminium alloy frame. The connecting cables of each channel are strictly cut to the same length. The local oscillator (LO) and calibration reference signals are generated by the separate single-channel DDS devices and fed into each channel by power splitters to maintain the consistency.

## 4. Typical Experimental Results 

Based on this prototype device, the verification experiment was carried out in Kunshan (120°57′ E, 31°30′ N), Jiangsu Province, China, from December 2017 to January 2018. The whole experiment was conducted respectively for two parts: one to carry out the MF sounding to verify the mesospheric sounding capability of MAI system, and the other to sound the ionosphere in the form of vertical sounding to verify the ionospheric sounding performance of MAI system. They will be described separately below.

### 4.1. Mesospheric Sounding

**Figure 13:**
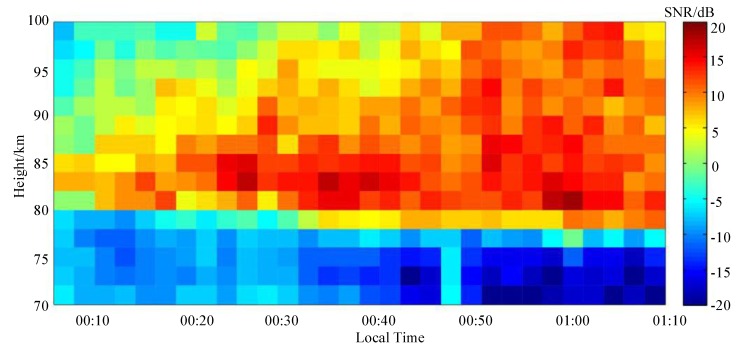
SNR map for mesospheric sounding at the local time of 00:08−01:08, 1 January 2018.

The sounding of the mesosphere was operated first. MAI system worked in a fixed frequency at 1.98 MHz. The number of coherent accumulations is 1024. As a representative example, Figure 13 shows a typical SNR map of the mesospheric sounding obtained by one receiving antenna. It can be seen that MAI system has successfully realized the MF radar’s ability to sound the mesosphere at a small transmitting power. The maximum SNR has reached more than 35 dB. The main altitude distribution of the signals’ reflection region is 75–100 km. And the electron density changes rapidly in time and space. 

By employing the FCA algorithm [4,5], the echo signals of the equilateral triangle antenna array can be used to estimate the drift velocity of the wind field. When the coordinates of the antennas of each vertex of the array is set to $\left(x_{1},y_{1}\right)$,$\left(x_{2},y_{2}\right)$,$\left(x_{3},y_{3}\right)$, and the signal time delay is τ, the correlation functions between the signals can be obtained: $\rho (\xi_{12},\eta_{12},\tau )$, $\rho (\xi_{23},\eta_{23},\tau )$ and $\rho (\xi_{13},\eta_{13},\tau )$, where $\xi_{ij}=x_{j}-x_{i}$, $\eta_{ij}=y_{j}-y_{i}$. Under the hypothetical condition that the correlation function ρ between the antenna signals is only related to the antenna spacing (ξ,η) and the signal time delay τ, its basic form can be expressed as a concentric ellipse cluster as the Equation (11).

(11) $$\rho (\xi ,\eta ,\tau )=\rho (A\xi^{2}+B\eta^{2}+C\tau^{2}+2H\xi \eta )$$

When the diffraction pattern of the sounding object has a drift velocity $\left(V_{x},V_{y}\right)$, Equation (11) can be rewritten to the Equation (12)

(12) $$\rho (\xi^{\prime },\eta^{\prime },\tau )=\rho (A{\left(\xi^{\prime }-V_{x}\tau \right)}^{2}+B{\left(\eta^{\prime }-V_{y}\tau \right)}^{2}+C\tau^{2}+2H(\xi^{\prime }-V_{x}\tau )\eta (\eta^{\prime }-V_{y}\tau ))$$

where $\left(\xi^{\prime },\eta^{\prime }\right)$ is defined as the Equation (13):

(13) $$\xi^{\prime }=\xi +V_{x}\tau ,\eta^{\prime }=\eta +V_{y}\tau$$

In terms of eliminating the coefficient C, the correlation function can be expressed by the coefficient parameters a,b,f,g,h as the Equation (14):

(14) $$\rho (\xi ,\eta ,\tau )=\rho (a\xi^{2}+b\eta^{2}+\tau^{2}+2f\xi \tau +2g\eta \tau +2h\xi \eta )$$

Define $\tau_{ij}^{}$ as the maximum delay time of $\rho (\xi_{ij},\eta_{ij},\tau )$, and then the Equation (15) should be satisfied:

(15) $$\frac{\partial \rho (\xi_{ij},\eta_{ij},\tau )}{\partial \tau }=0,\tau_{ij}^{}=-f\xi_{ij}-g\eta_{ij}$$

Define $\tau_{ij}^{\prime }$ to express the delay time which is determined by $\rho (\xi_{ij},\eta_{ij},0)=\rho (0,0,\tau_{ij}^{\prime })$, the Equation (16) can be obtained:

(16) $$\{\begin{array}{c} \rho (\xi_{ij},\eta_{ij},0)=\rho (a\xi_{ij}^{2}+b\eta_{ij}^{2}+0+2f\xi_{ij}0+2g\eta_{ij}0+2h\xi_{ij}\eta_{ij})=\rho (a\xi_{ij}^{2}+b\eta_{ij}^{2}+2h\xi_{ij}\eta_{ij})\\ \rho (0,0,\tau_{ij}^{\prime })=\rho (a0^{2}+b0^{2}+\tau_{ij}^{\prime 2}+2f0\tau_{ij}^{\prime }+2g0\tau_{ij}^{\prime }+2h00)=\rho (\tau_{ij}^{\prime 2})\\ \tau_{ij}^{\prime 2}=a\xi_{ij}^{2}+b\eta_{ij}^{2}+2h\xi_{ij}\eta_{ij} \end{array}$$

Then, when the equation $\rho (\xi_{ij},\eta_{ij},\tau_{ij})=\rho (0,0,\tau_{ij}^{\prime \prime })$ is established, the delay time $\tau_{ij}^{\prime \prime }$ can be determined by the Equation (17):

(17) $$\tau_{ij}^{\prime \prime 2}=\tau_{ij}^{\prime 2}-\tau_{ij}^{2}$$

Based on Equations (15)–(17), the coefficients parameters a,b,f,g,h can be solved. Then, we can use these coefficients to calculate the velocity of the wind field $\left(V_{z},V_{m}\right)$ as Equation (18):

(18) $$\{\begin{array}{c} V_{z}=V_{x}/2=(hg-bf)/2(ab-h^{2})\\ V_{m}=V_{y}/2=(hf-ag)/2(ab-h^{2}) \end{array}$$

**Figure 14:**
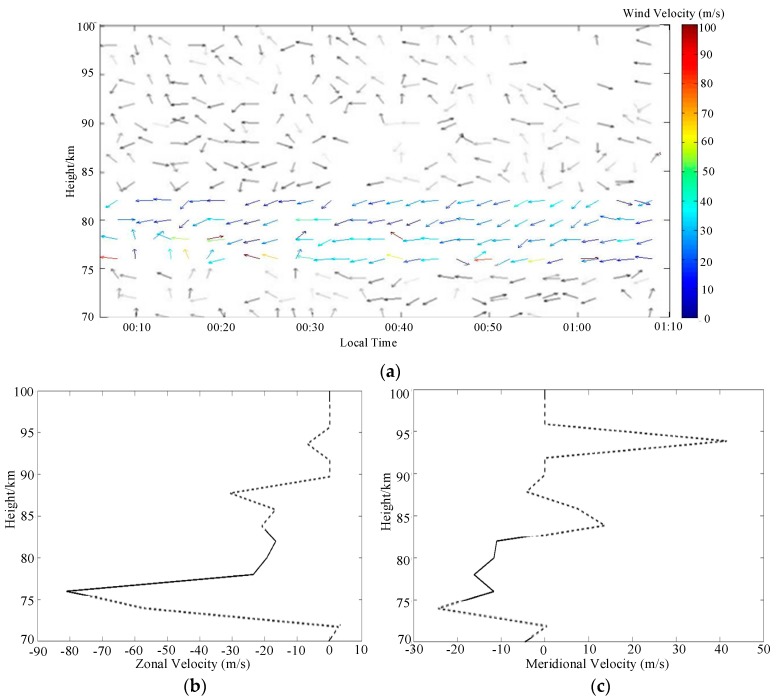
Wind field inversion results by FCA at the local time of 00:08-01:08, 1 January, 2018: (**a**) the temporal and spatial characteristics of wind field. Beyond 75−83 km, it is marked in gray for the less of accuracy (**b**) the zonal profile at 00:50; (**c**) the meridional profile at 00:50. Beyond 75−83 km, they are also marked as dashed lines.

Based on the signals of the three vertices of the antenna array, Figure 14a shows the wind field estimation result during the same time period of Figure 13. The direction of the arrow represents the direction of the wind field. In the range of 75–83 km, MAI system obtained a relatively stable wind field estimation. This is also consistent with the signal energy distribution in Figure 13. The range of velocity distribution is mainly between 20–60 m/s. But beyond this range, where are marked in grey, it becomes less accurate, which may be caused by the low SNR or the effect of the oblique echoes in 00:40–01:00 LT. Figure 14b,c are the zonal and meridional wind profiles at 00:50 LT. Beyond 75–83 km, the profiles are also shown as dashed lines. It can be seen that the velocities are distributed in tens of meters per second. At the height of 75–83 km, the zonal wind is mainly between–18 m/s and –23 m/s, which is relatively stable. Conversely, the meridional wind varies rapidly with the height, from –5 m/s to –12 m/s in the range of 75–83 km.

In addition to the wind field, the sounding data of MAI system can also be used to inverse the electron density of the low ionosphere. For MF/HF radar, DAE algorithm is often applied to calculate the electron density [35]. According to DAE algorithm, when the ionosphere is modeled into a myriad of thin layers, the transmittance of the hth layer and the reflectivity of the current reflecting Hth layer can be defined as exp[−ΔhK(h)] and R(H). Δh is a small range separation of two adjacent layers. By ignoring the secondary reflection, the amplitude A(H) of hth layer’s echo can be expressed as Equation (19), where Y is the other loss besides the ionosphere.

(19) $$A(H)=Y\exp\left[-\Delta h\sum_{h=1}^{H}K(h)\right]R(H)\exp\left[-\Delta h\sum_{h=1}^{H}K(h)\right]=Y\cdot R(H)\exp\left[-2\Delta h\sum_{h=1}^{H}K(h)\right]$$

where K(h) is the absorption coefficient of the hth layer. 

In the case of the MAI system, the two orthogonally erected antennas at each vertex of the array make it possible to be used for receiving the O and X wave signals alternately. Since there are different transmittance and reflectivity parameters for the O and X echo signals, Equation (19) can be specifically written as Equation (20):

(20) $$\{\begin{array}{c} A_{O}(H)=Y_{O}R_{O}(H)\exp\left[-2\Delta h\sum_{h=1}^{H}K_{O}(h)\right]\\ A_{X}(H)=Y_{X}R_{X}(H)\exp\left[-2\Delta h\sum_{h=1}^{H}K_{X}(h)\right] \end{array}$$

When it is assumed that $Y_{O}=Y_{X}$, then Equation (21) can be obtained:

(21) $$\frac{A_{x}(H)}{A_{o}(H)}=\frac{R_{x}(H)}{R_{o}(H)}\exp\left[-2\Delta h\sum_{h=1}^{H}\left(K_{x}(h)-K_{o}(h)\right)\right]$$

When the logarithms are taken simultaneously on the left and right sides of Equation (21), it can be transformed into Equation (22):

(22) $$\{\begin{array}{c} \ln\left[\frac{A_{x}(H)}{A_{o}(H)}\right]=\ln\left[\frac{R_{x}(H)}{R_{o}(H)}\right]-2\Delta h\sum_{h=1}^{H}\left(K_{x}(h)-K_{o}(h)\right)\\ \ln\left[\frac{A_{x}(H)}{A_{o}(H)}\right]-\ln\left[\frac{A_{x}(H-1)}{A_{o}(H-1)}\right]=\ln\left[\frac{R_{x}(H)}{R_{o}(H)}\right]-\ln\left[\frac{R_{x}(H-1)}{R_{o}(H-1)}\right]-2\Delta h\left[K_{x}(H)-K_{o}(H)\right] \end{array}$$

In practical calculation, the reflection coefficient $R_{O/X}(H)$ can be obtained from Equation (23):

(23) $$R_{O/X}(H)={\left[\frac{{\left\{\frac{\omega -\omega_{L}}{v_{m}(H)}C_{3/2}(\frac{\omega -\omega_{L}}{v_{m}(H)})\right\}}^{2}+{\left\{\frac{5}{2}C_{5/2}(\frac{\omega -\omega_{L}}{v_{m}(H)})\right\}}^{2}}{{\left\{\frac{\omega +\omega_{L}}{v_{m}(H)}C_{3/2}(\frac{\omega +\omega_{L}}{v_{m}(H)})\right\}}^{2}+{\left\{\frac{5}{2}C_{5/2}(\frac{\omega +\omega_{L}}{v_{m}(H)})\right\}}^{2}}\right]}^{1/2}$$

where ω is the angular frequency of the sounding signal. $\omega_{L}$ is the longitudinal component of angular cyclotron frequency, which can be calculated according to the magnetic dip based on International Geomagnetic Reference Field (IGRF). $v_{m}(H)$ is the collision frequency of Hth layer and can be calculated relay to the atmospheric pressure profile according to International Reference Atmosphere (CIRA) of the Committee on Space Research (COSPAR) [36]. $C_{3/2}()$ and $C_{5/2}()$ can refer to [37].

When F(H) is defined as Equation (24), after calculating F(H), electron density profile of the corresponding height at Hth layer N(H) can be obtained by substituting Equation (24) into Equation (22) [36].

(24) $$F(H)=\frac{2\left[K_{x}(H)-K_{O}(H)\right]\Delta h}{N(H)}$$

Specifically, F(H) can be calculated by Equations (25) and (26).

(25) $$\{\begin{array}{c} F_{O}(H)=\frac{5}{4}\frac{e^{2}}{m\varepsilon_{0}cv_{m}(H)}C_{5/2}(\frac{\omega +\omega_{L}}{v_{m}(H)})\\ F_{X}(H)=\frac{5}{4}\frac{e^{2}}{m\varepsilon_{0}cv_{m}(H)}C_{5/2}(\frac{\omega -\omega_{L}}{v_{m}(H)}) \end{array}$$

(26) $$F(H)=2\left[F_{X}(H)-F_{O}(H)\right]\Delta h$$

where $e,m,\varepsilon_{0}$ represent the electric quantity, electronic mass and dielectric constant respectively.

Then, the electron density profile of the corresponding height at Hth layer N(H) can be expressed as Equation (27) [36]

(27) $$N(H)=\frac{\left\{\ln\left[\frac{R_{X}(H)}{R_{O}(H)}\right]-\ln\left[\frac{R_{X}(H-1)}{R_{O}(H-1)}\right]\right\}-\left\{\ln\left[\frac{A_{X}(H)}{A_{O}(H)}\right]-\ln\left[\frac{A_{X}(H-1)}{A_{O}(H-1)}\right]\right\}}{F(H)}$$

**Figure 15:**
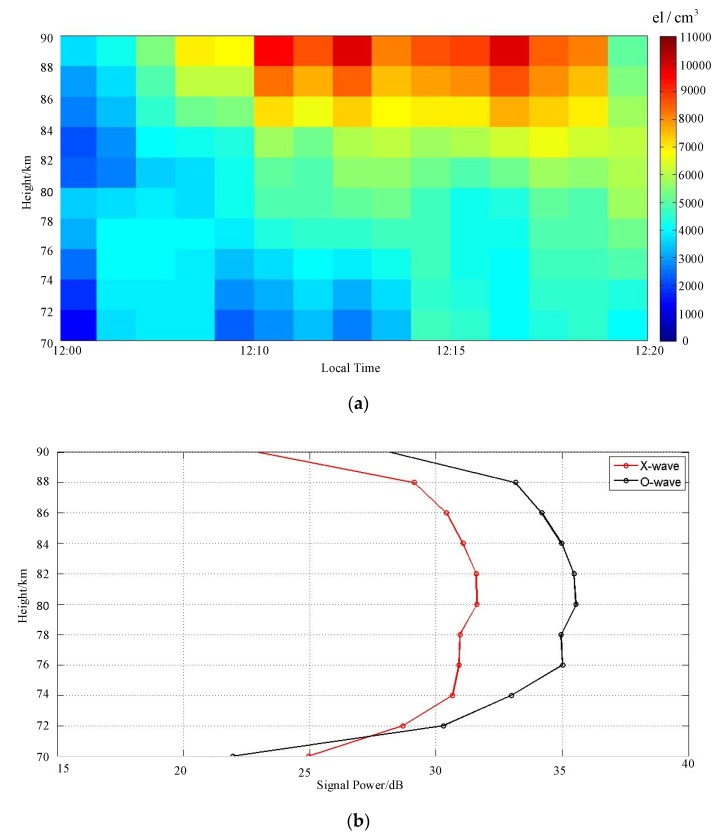
(**a**) the electron density inversion results of DAE algorithm at the local time of 11:00−12:00, 8 January 2018. (**b**) shows the signal power of X-wave mode and O-wave mode at the local time of 11:15, 8 January 2018.

Figure 15a shows a typical result of the inversion based on the DAE algorithm. Figure 15b shows a typical signal power distribution with height. Considering that the D layer of the ionosphere has a low electron density at night, which is difficult to reflect the MF signals, we chose the sounding data at noon for electron density inversion. From 70 km onwards, the electron density increases with the height. During the observation period, the electron density also increased slightly over time.

### 4.2. Ionospheric Sounding

**Figure 16:**
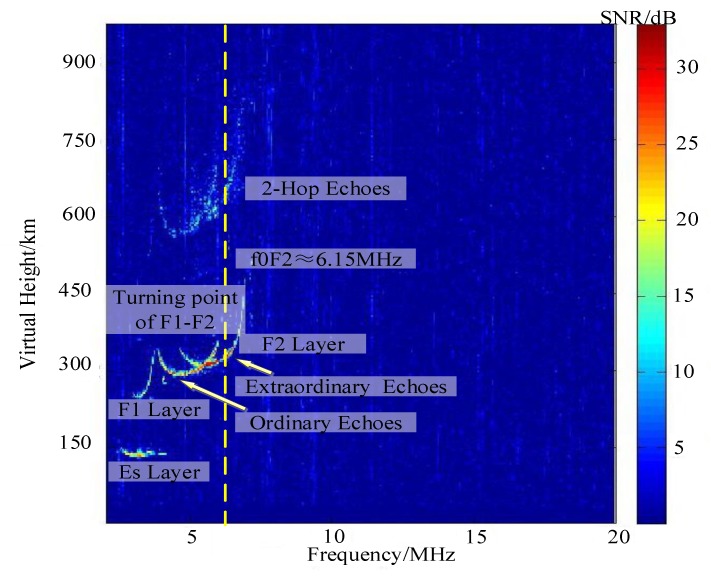
An example of vertical sounding ionogram at the local time of 17:50, 5 January 2018.

The sounding of the ionosphere adopts the single-transmit and single-receive mode. Switching from the mesospheric sounding mode requires a two-wire dipole progressive-wave antenna to be connected to the transmitting channel as a transmitting antenna to match a larger bandwidth. In the ionosonde mode, DDS upconverter module converts the output frequency according to instructions transmitted by USB bus, and LO also changes synchronously. The frequency sweeps in a range from 2 MHz to 20 MHz with a step of 50 kHz. At each frequency point, 32 coherent accumulations were performed, and the residence time was 0.26 s. In the vertical sounding mode, the MAI system obtains the ionogram shown in Figure 16. At the local time of 17:50, 5 January, 2018, the maximum SNR is about 30 dB. The ordinary (O) and the extraordinary (X) echoes can be clearly observed. This ionogram contains the echos of the Sporadic-E (Es) layer, F1 layer, F2 layer and the 2-hop echoes of the F2 layer. In the height distribution, the virtual height of the Es layer mainly distributes near 120 km with the corresponding delay is about 0.83 ms. The virtual heights of the F1 and F2 layers are 210–305 km and 260–320 km, respectively. There is a clear turning point between them. Further, the critical frequency of F2 layer is about f0F2 = 6.15 MHz. This ionogram obtained by MAI system is typical and similar to the traditional ionograms as well.

For the ionogram, we use the quasi-parabolic segments (QPS) model to invert the electron density. In QPS model, the E, F1, and F2 layers are represented by a single quasi-parabolic (QP) model [38,39,40,41,42], while the transition regions of the E-F1 and F1-F2 are represented by a reverse QP model. Therefore, the whole electron density profile can be described as in Equation (28).

$$N_{F2}=a_{F2}-b_{F2}{\left(1-\frac{r_{F2}}{r}\right)}^{2}\begin{array}{cc} & F2\begin{array}{cc} & layer \end{array} \end{array};\;a_{m}=N_{m},b_{m}=N_{m}(r_{m}/y_{m});a_{jFF}=a_{F1},r_{jFF}=r_{F1}$$

$$N_{F_{1}}=a_{F_{1}}-b_{F_{1}}{\left(1-\frac{r_{F_{1}}}{r}\right)}^{2}\begin{array}{cc} & F1\begin{array}{cc} & layer \end{array} \end{array};\;b_{jFF}=-\frac{b_{F2}r_{F2}(1-r_{F2}/r_{cF2})}{r_{F1}(1-r_{F1}/r_{cF2})}$$

$$N_{E}=a_{E}-b_{E}{\left(1-\frac{r_{E}}{r}\right)}^{2}\begin{array}{cc} & E\begin{array}{cc} & layer \end{array} \end{array};\;r_{cF2}=-\frac{b_{F2}r_{F2}(r_{F2}/r_{F1}-1)}{a_{F2}-a_{F1}+b_{F2}(r_{F2}/r_{F1}-1)}$$

$$N_{jFE}=a_{jFE}+b_{jFE}{\left(1-\frac{r_{jFE}}{r}\right)}^{2}\begin{array}{cc} & E-F1 \end{array};\;a_{jFE}=a_{E},r_{jFE}=r_{E},b_{jFE}=-\frac{b_{F1}r_{F1}(1-r_{F1}/r_{cF1})}{r_{E}(1-r_{E}/r_{cF1})}$$

(28) $$N_{jFF}=a_{jFF}+b_{jFF}{\left(1-\frac{r_{jFF}}{r}\right)}^{2}\begin{array}{cc} & F1-F2 \end{array};\;r_{cF1}=-\frac{b_{F1}r_{F1}(r_{F1}/r_{E}-1)}{a_{F1}-a_{E}+b_{F1}(r_{F1}/r_{E}-1)}$$

The calculation of the whole electron density profile is determined by nine parameters: $N_{m}F_{2}$ is the peak of electron density in the F2 layer, $r_{m}F_{2}$ is the peak distance from the geocentric distance, $y_{m}F_{2}$ is half thickness of the F2 layer. $N_{m}F_{1}$, $r_{m}F_{1}$, $y_{m}F_{1}$, $N_{m}E$, $r_{m}E$, $y_{m}E$, are the corresponding parameters of the F1 layer and E layer, respectively. $a_{m}=N_{m}$ is the peak electron density of the corresponding layer, $r_{m}$ is the peak distance from the centrosphere, $y_{m}$ is half thickness of the corresponding layer. $b_{m}$ is the intermediate parameter of the calculation.

**Figure 17:**
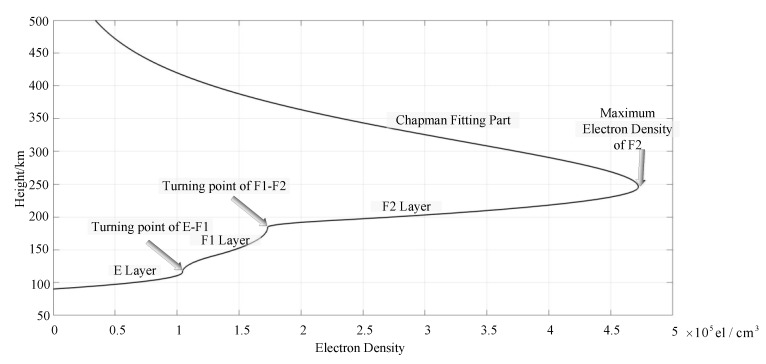
Electron density profile at the local time of 17:50, 5 January 2018.

According to the QPS model, Figure 17 shows the electron density profile obtained from the inversion of the ionogram of Figure 16. The electron density profile above the peak height is fitted by Chapman model [42]. As can be seen from Figure 17, the profile of the inversion layer contains the E, Fl and F2 layer with obvious turning points between the adjacent layers. The boundary between the E layer and the F1 layer is at the true height of 113.5 km. For the F1 and F2 layers, the boundary is at 185.5 km. The true peak height of the critical frequency is 247.1 km, with an electron density of $4.722\times 10^{5}\;\mathrm{el}/{\mathrm{cm}}^{3}$. Since the Es layer does not exist conventionally, the inversion for the Es layer is included in the program.

According to the above experimental results, the design concept and engineering value of the MAI system are verified. On the same hardware platform, the MAI system combines the function of the MF radar for the sounding of the lower ionosphere and the ionospheric sounding function of the ionosonde. Through the inversions of the sounding data, the wind field of the mesosphere, the profiles of the electron density in the lower ionosphere, and the electron density above the conventional ionosphere E layer can be obtained. If these results are used scientifically in future research, it will be very promising to study the physical characteristics of the lower ionosphere and ionosphere.

## 5. Conclusions

In this paper, we propose a new type of sounding system which integrates the MF radar’s sounding function of the mesosphere (which also belongs to the lower ionosphere) and the ionosonde’s sounding function of the ionosphere above E layer into one hardware platform which is denoted as the middle atmosphere-ionosphere (MAI) system. It breaks through the monopulse mode adopted by traditional MF radar. MAI system innovatively adopts the pseudo-random phase code modulation on the waveform design. Relying on the pulse compression, an extra correlation gain would be obtained, and thus the transmitting power can be reduced to simplify the equipment and consequently the cost. The employment of the complementary code sequences also enables MAI system to have a strong anti-jamming capability. For the antennas employed in MAI system, unlike the traditional MF radars, a mode of one transmitting and six receiving is adopted for the mesospheric (lower ionospheric) sounding. The progressive-wave antennas are employed for receiving, which miniaturizes the array structure, reduces the difficulty of erection, and satisfies the large bandwidth for the ionospheric sounding. Although the efficiency may be lost, the waveform gain would compensate for this. Using the developed prototype device, we conducted a verification experiment. Based on the experimental data, using FCA and DAE algorithms, the drift velocities of the wind field and the electron density profiles of the lower ionosphere are estimated or calculated. By sweeping in high frequency band, MAI system conducted the ionospheric vertical sounding. After inversing with the QPS model based on the ionogram, the ionospheric electron density profile could be acquired. The successful development of MAI system makes it possible to sound the two regions on the same platform at the same location. This is of significance to the study of the coupling characteristics and physical processes of them. With the further improvement of experiments, the scientific research based on MAI system will be more desirable.

For the current MAI system, in order to better carry out future research tasks, we think that we need to improve some of its shortcomings. Firstly, the current signal quality needs to be strengthened. Both the wind field estimation and the low ionospheric electron density inversion have some data points missing. While this will not affect the macroscopic spatial and temporal analysis, the performance details need improvements. Secondly, at present, the electron density profiles of the lower ionosphere and the upper ionosphere have not been connected in series. Further, if we combine the sounding results together, the continuous electron density profiles from the D layer to the F layer is very promising. The cause of the abnormal phenomenon of the electron density at lower ionosphere is also worthy of in-depth study. Finally, the current two sounding functions are time-independent. If the time-sharing operation is modified to offer a continuous mode, the sounding results would be more scientifically meaningful. 

## Figures and Tables

**Table 1:** The main technical indicators of MAI system.

Technical Indicators	Values
System function	Mesospheric/Ionospheric sounding
Working frequency	Mesospheric: 1.98 MHz/ Ionospheric: 2−20 MHz
Sounding range	Mesospheric: 70−100 km/ Ionospheric: 100−800 km
Peak power	Mesospheric: 6 Kw/ Ionospheric: ≤1 Kw
Waveform	Interpulse coding waveform
Duty cycle	5%
Height resolution	1.98 km
Time resolution	Mesospheric: ≤2 min/ Ionospheric: ≤4 min
Sounding mode	Mesospheric: Fixed Frequency/Ionospheric: Frequency sweeping
Sounding Direction	Vertical upward

**Table 2:** The code sequences of 16-bit bi-phase complementary code.

Sequence	Values
The positive sequence A ($a_{1}\cdots a_{L},L=16$)	1101_0001_0111_1011
The complement sequence B ($b_{1}\cdots b_{L},L=16$)	0010_1110_0111_1011

**Table 3:** The variation of the transmitting antenna characteristics with the erection height.

Parameters	H = 2 m	H = 3 m	H = 4 m	H = 5 m	H = 6 m
Directivity Gain (dB)	8.5	8.51	8.52	8.54	8.55
Beamwidth (°)	67.17	67.28	67.19	67.22	67.27
VSWR.	1.15	1.12	1.21	1.28	1.3

**Table 4:** The variation of the receiving antenna characteristics with the erection height.

Parameters	H = 4 m	H = 8 m	H = 12 m	H = 20 m	H = 25 m
Directivity gain(dB)	7.76	7.99	8.24	8.33	8.37
Beamwidth(°)	70	70.26	70.92	72.86	74.59
